# The Emerging Role and Mechanism of E2/E3 Hybrid Enzyme UBE2O in Human Diseases

**DOI:** 10.3390/biomedicines13051082

**Published:** 2025-04-29

**Authors:** Qian Cheng, Zuyin Li, Yongjian Li, Lei Chen, Dingbao Chen, Jiye Zhu

**Affiliations:** 1 Department of Hepatobiliary Surgery, Peking University People’s Hospital, Beijing 100044, China; chengqian@bjmu.edu.cn (Q.C.); lizuyin2018@163.com (Z.L.); yongjianli@pku.org.cn (Y.L.); chenlei@pkuph.edu.cn (L.C.); 2 Peking University Institute of Organ Transplantation, Peking University, Beijing 100044, China; 3 Beijing Key Laboratory of HCC and Liver Cirrhosis, Peking University People’s Hospital, Beijing 100044, China; 4 Department of Pathology, Peking University People’s Hospital, Beijing 100044, China; cdingbao@163.com

**Keywords:** UBE2O, solid tumors, hematologic malignancies, substrates, Alzheimer’s disease, metabolic diseases, substrates

## Abstract

The ubiquitin–proteasome system (UPS) plays a pivotal role in determining protein fate, regulating signal transduction, and maintaining cellular homeostasis. Protein ubiquitination, a key post-translational modification, is orchestrated by the sequential actions of three primary enzymes, ubiquitin-activating enzyme (E1), ubiquitin-conjugating enzyme (E2), and ubiquitin protein ligase (E3), alongside the regulatory influence of deubiquitinases (DUBs) and various cofactors. The process begins with E1, which activates ubiquitin molecules. Subsequently, E2 receives the activated ubiquitin from E1 and transfers it to E3. E3, in turn, recognizes specific target proteins and facilitates the covalent attachment of ubiquitin from E2 to lysine residues on the target protein. Among the E2 enzymes, ubiquitin-conjugating enzyme E2O (UBE2O) stands out as a unique E2–E3 hybrid enzyme. UBE2O directly mediates the ubiquitination of a wide array of substrates, including 5′-AMP-activated protein kinase catalytic subunit alpha-2 (AMPKα2), MAX interactor 1 (Mxi1), and v-maf musculoaponeurotic fibrosarcoma oncogene homolog (c-Maf), among others. In this narrative review, we will explore the structural characteristics of UBE2O and elucidate its molecular functions. Additionally, we will summarize recent advancements in understanding the role of UBE2O in various tumors, Alzheimer’s disease (AD), and metabolic diseases. Finally, we will discuss the potential of targeting UBE2O as a novel therapeutic strategy for the treatment of human diseases.

## 1. Introduction

The post-translational modification of proteins through ubiquitination plays a critical role in maintaining cellular homeostasis by regulating protein degradation, localization, and activity, thereby ensuring proper cellular function and balance [1,2]. Ubiquitination regulates a broad spectrum of essential biological processes, including DNA repair, gene transcription, intracellular protein trafficking, glucose and lipid metabolism, angiogenesis, and inflammatory responses [3,4,5,6,7,8,9]. The dysregulation of ubiquitination is closely associated with the pathogenesis and progression of various diseases, such as malignant tumors, neurodegenerative disorders, cardiovascular diseases, and autoimmune conditions.

The ubiquitin–proteasome system (UPS) comprises ubiquitin-activating enzyme E1, ubiquitin-conjugating enzyme E2, ubiquitin ligase E3, proteasomes, deubiquitinases (DUBs), and various cofactors [9]. E1 utilizes adenosine triphosphate (ATP) to activate ubiquitin and subsequently transfers it to E2. E2 then interacts with specific E3 ligases, which facilitate the transfer of ubiquitin to the target protein. Ultimately, the ubiquitinated substrate is recognized and degraded by the 26S proteasome [9]. In addition, deubiquitinases counteract the function of ubiquitin ligases by removing ubiquitin from substrate proteins, thereby regulating the ubiquitination process [3]. Cofactors involved in this system include valosin-containing protein (p97/VCP), the transcriptional coactivator β-catenin, adenosine 5′-monophosphate-activated protein kinase (AMPK)-related kinases, and others.

Ubiquitin-Conjugating Enzyme E2O (UBE2O) is a dual-function ubiquitin-modifying enzyme possessing both E2 and E3 enzymatic activities, and it has garnered significant attention in recent years due to its critical roles in various diseases. This article reviews the molecular functions of UBE2O and its underlying mechanisms in cancer, metabolic diseases, and age-related diseases. We hope this review will stimulate further research to enhance our understanding of UBE2O-related diseases and accelerate the discovery of novel therapeutic strategies targeting UBE2O in human diseases.

## 2. Molecular Characteristics and Functions of UBE2O

**Figure 1:**
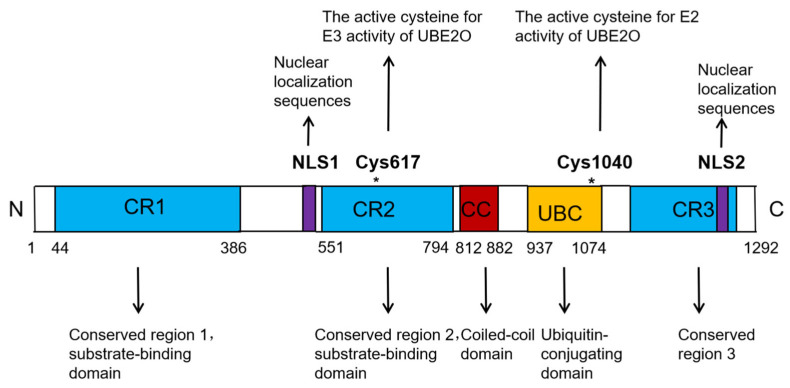
Domain architecture of UBE2O. The UBE2O protein (1292 amino acids) comprises three conserved regions (CR1, CR2, CR3), a ubiquitin-conjugating (UBC) domain, and two nuclear localization sequences (NLSs). The CR1 and CR2 domains mediate substrate binding, whereas the UBC domain facilitates interactions with multiple E3 ligases. Catalytic activation sites are localized to Cys-1040 (E2) and Cys-617 (E3) residues, respectively.

UBE2O is a unique member of the E2 ubiquitin-conjugating enzyme family. Unlike the majority of E2 enzymes, which typically have a molecular weight ranging between 20 and 30 kDa, UBE2O is significantly larger, with a molecular weight of 141 kDa. The UBE2O protein consists of 1292 amino acids and contains several key functional domains, including three conserved regions (CR1, CR2, and CR3), a coiled-coil (CC) domain, one ubiquitin-conjugating (UBC) domain, and two nuclear localization sequences (NLSs) (Figure 1) [10,11,12]. The UBC domain is responsible for interacting with multiple E3 ligases, while the CR1 and CR2 domains are involved in substrate binding (Figure 1) [10,11,12]. Within the human UBE2O protein, Cys1040, located in the UBC domain, is critical for its E2 activity, whereas Cys617, situated in the CR2 domain, is essential for its E3 activity (Figure 1) [10,11,12]. Notably, numerous studies have demonstrated that UBE2O can ubiquitinate certain substrates independently of other E3 ligases. This dual functionality highlights UBE2O’s unique role as both an E2 ubiquitin-conjugating enzyme and an E3 ubiquitin ligase, classifying it as an E2/E3 hybrid enzyme [10,13].

UBE2O is capable of mediating the mono-ubiquitination, multi-monoubiquitination, and polyubiquitination of various substrates, thereby performing diverse biological functions. For instance, UBE2O acts as a positive regulator of bone morphogenetic protein 7 (BMP7) signaling by mediating the monoubiquitination of SMAD6, which plays a role in regulating adipogenesis [13,14]. During the maturation of red blood cells, the degradation of ribosomal proteins is one of the key steps. UBE2O marks ribosomal proteins for degradation by the proteasome through multi-monoubiquitination. Studies have shown that the absence of UBE2O leads to the accumulation of ribosomal proteins in reticulocytes, thereby affecting the normal maturation of red blood cells [15]. UBE2O also functions as a ubiquitinase for the AMP-activated protein kinase α2 catalytic subunit (AMPKα2), facilitating its polyubiquitination and degradation. This process activates the mechanistic/mammalian target of rapamycin complex 1 (mTORC1) signaling pathway, ultimately driving the transformation and metastasis of breast cancer [16].

As highlighted above, UBE2O functions as an E2/E3 hybrid ubiquitin ligase. Interestingly, UBE2O also exhibits non-enzymatic functions, such as mediating changes in subcellular localization and protein interactions. For example, UBE2O promotes the cytoplasmic retention of breast cancer susceptibility gene 1(BRCA1)-associated protein 1 (BAP1) by monoubiquitinating its nuclear localization signal (NLS) region, thereby preventing its nuclear entry. This inhibition affects various nuclear processes involving BAP1, including gene expression regulation, cell cycle progression, and DNA repair [17,18]. Furthermore, UBE2O binds to TNF receptor associated factor 6 (TRAF6) and inhibits its K63- and K48-linked polyubiquitination. This disruption impairs the interaction between TRAF6 and myeloid differentiation primary response protein 88 (MyD88), leading to the suppression of the TRAF6-mediated activation of the nuclear factor kappa-light-chain-enhancer of activated B cells (NF-κB) signaling pathway [19].

In recent years, studies have demonstrated that UBE2O plays a significant role in carcinogenesis and tumor progression. In this review, we summarize the contributions of UBE2O to tumor development and elucidate the mechanisms by which this signaling network regulates oncogenic processes. We hope this review will stimulate further research to enhance our understanding of UBE2O-mediated tumorigenesis and accelerate the discovery of novel therapeutic strategies targeting UBE2O in cancer treatment.

## 3. Roles and Mechanisms of UBE2O in Cancer

### 3.1. The Function and Molecular Mechanism of UBE2O in Solid Tumors

Solid tumors represent a major global health burden due to their complex etiology and resistance to conventional therapies. The ubiquitin–proteasome system (UPS), a critical regulator of cellular processes such as protein degradation, DNA repair, and cell cycle control, has emerged as a key focus in cancer research. UBE2O, an E2 ubiquitin-conjugating enzyme, plays a tumor-promoting role in multiple solid tumors, including breast cancer, prostate cancer, hepatocellular carcinoma (HCC), and non-small cell lung cancer (NSCLC), by driving tumor progression [16,20,21,22,23,24,25,26].

#### 3.1.1. The Role of UBE2O in Breast and Prostate Cancer via the AMPKα2/mTORC1/HIF1α Signaling Pathway

In 2017, Vila et al. revealed the crucial role of UBE2O in breast and prostate cancer using Ube2o knockout (KO) mouse models [16]. In breast cancer, *Ube2o^−/^^−^* mice crossed with the MMTV-PyVT (mouse mammary tumor virus–polyoma virus middle T antigen) model exhibited delayed tumor onset, reduced tumor volume/weight, and fewer lung metastases compared with controls. Similarly, in the TRAMP (transgenic adenocarcinoma mouse prostate) model, *Ube2o^−/^^−^* mice had a lower incidence of prostate tumors, reduced tumor burden, and no liver/lymph node metastases [16]. These findings highlight the role of UBE2O in promoting tumor growth and metastasis. In addition, UBE2O also enhances epithelial–mesenchymal transition (EMT) and cancer stem cell (CSC) characteristics in breast cancer, which are crucial for invasion, metastasis, and tumor recurrence [16,20].

In terms of metabolic reprogramming, UBE2O drives metabolic reprogramming, which is a hallmark of cancer. Breast cancer cells deficient in UBE2O show reduced glycolytic metabolites (such as glucose-6-phosphate and fructose-6-phosphate), smaller cell size, and impaired purine/amino acid biosynthesis—key processes that support rapid proliferation [16]. These cells also exhibit decreased glucose consumption and lactate production, reflecting an “anti-Warburg effect”. The Warburg effect—preferential glycolysis under oxygen-rich conditions—is suppressed in UBE2O knockout cells, further emphasizing the role of UBE2O in maintaining cancer metabolism [16]. Similar metabolic changes have been observed in prostate cancer, indicating that this mechanism is conserved across different tumor types [16].

Regarding the molecular mechanism, UBE2O acts as an E2/E3 hybrid ubiquitin ligase targeting AMPKα2. UBE2O directly ubiquitinates AMPKα2 (but not AMPKα1) via K48-linked polyubiquitination at lysine 470 (K470), which is dependent on its catalytic cysteine residue (C1040) [16]. This modification triggers proteasomal degradation. The degradation of AMPKα2 by UBE2O relieves the inhibition of mTORC1 [16]. Normally, AMPK inhibits mTORC1 by phosphorylating tuberous sclerosis complex subunit 2 (TSC2). The loss of AMPKα2 driven by UBE2O activates mTORC1, enhancing glycolysis, biosynthetic pathways, and tumor growth. The overexpression of UBE2O increases glucose uptake and lactate production, while its deficiency reverses these effects, which is consistent with the metabolic roles of mTORC1. The activation of mTORC1 stabilizes hypoxia inducible factor 1 alpha (HIF1α) by promoting its translation (via eukaryotic translation initiation factor 4E-binding protein 1(4E-BP1)/eukaryotic translation initiation factor 4E (eIF4E) inhibition) and phosphorylation. HIF1α upregulates the genes involved in glycolysis, angiogenesis, and proliferation (such as vascular endothelial growth factor A (VEGFA) and glucose transporter 1 (GLUT1). The overexpression of UBE2O amplifies HIF1α targets, while its deficiency suppresses these targets, thereby inhibiting tumor progression [16].

In terms of clinical significance, data from The Cancer Genome Atlas (TCGA) show that UBE2O is overexpressed in approximately 20% of breast cancers, which is associated with the amplification of 17q25. Tissue microarrays confirm that UBE2O expression levels are higher in 72 breast tumors than in normal tissues. High UBE2O expression is correlated with poor prognosis (shorter overall/recurrence-free survival), larger tumor size, lymph node metastasis, higher histological grade, HER2 positivity, and a higher prevalence of the triple-negative breast cancer (TNBC) subtype [16]. In prostate cancer, UBE2O expression is upregulated, negatively correlated with AMPKα2, and positively correlated with mTOR/HIF1α. High UBE2O expression is associated with poor prognosis, larger tumor volume, higher Gleason score, lymph node/distant metastases (such as to the liver and lung), and aggressive molecular subtypes [16].

#### 3.1.2. UBE2O Promotes the Progression of Hepatocellular Carcinoma (HCC)

Multiple studies have demonstrated that UBE2O protein expression is significantly elevated in HCC tissues compared to adjacent non-tumorous tissues [22,23,24,25]. This upregulation has also been confirmed in HCC cell lines (HCCLM3, MHCC97H, HepG2, Hep3B, and Huh7) relative to the normal liver cell line MIHA [24]. Clinically, high UBE2O expression correlates with poor prognosis in HCC patients, advanced tumor stage, high histological grade, and venous infiltration [22,23,24,25]. Functionally, UBE2O promotes HCC cell proliferation, migration, and invasion both in vitro and in vivo [22,23,24,25]. Notably, UBE2O-knockout mice exhibit resistance to diethylnitrosamine (DEN)-induced hepatocarcinogenesis [22].

To identify potential UBE2O substrates, Ma et al. performed immunoprecipitation-mass spectrometry (IP-MS) screening in HepG2, Huh7, and HEK293-T cell lines [22]. Three candidates emerged—hydroxyacyl-CoA dehydrogenase (HADHA), nascent polypeptide-associated complex subunit alpha (NACA), and solute carrier family 25 member 3 (SLC25A3). Among these, HADHA—a mitochondrial enzyme linked to fatty acid β-oxidation and implicated in multiple cancers [27,28,29]—showed reduced expression in poorly differentiated HCC, suggesting its tumor-suppressive role. UBE2O physically interacts with HADHA, recognizing lysine 129 to mediate K48-linked polyubiquitination and subsequent proteasomal degradation, thereby suppressing HADHA at the post-transcriptional level. As HADHA catalyzes critical steps in fatty acid β-oxidation (converting fatty acids to acetyl-CoA for energy production), its loss shifts HCC metabolism toward lipogenesis, fueling tumor proliferation and metastasis.

In breast carcinogenesis, UBE2O activates the mTOR signaling pathway by facilitating the ubiquitination-mediated degradation of AMPKα2 [16]. This regulatory mechanism has been experimentally validated in hepatocellular carcinoma (HCC) by Shi et al. The genetic knockdown of UBE2O significantly enhances AMPKα2 protein stability, whereas its overexpression reduces AMPKα2 levels and enhances mTOR phosphorylation (p-mTOR), thereby promoting the transcriptional activation of downstream target genes (including MYC, Cyclin D1, HIF1α, and sterol regulatory element-binding protein 1/SREBP1) [28]. Notably, the specific silencing of AMPKα2 effectively abolishes the mTOR pathway inhibition triggered by UBE2O depletion. Additional experiments have demonstrated that the pharmacological inhibition of mTOR with rapamycin (RAPA) not only blocks the UBE2O-driven upregulation of p-mTOR, but also markedly suppresses the proliferative capacity and migratory potential of HCC cells [24].

Despite advances, HCC prognosis remains poor due to late diagnosis and drug resistance. Interferon-α (IFN-α)—a cytokine with antitumor and immunomodulatory properties—is limited by therapeutic resistance, and is partly mediated by ubiquitination-dependent protein degradation. In vitro, UBE2O knockdown enhances IFN-α sensitivity, suppressing HCC cell proliferation, migration, and colony formation. In vivo, UBE2O-deficient xenografts exhibit reduced tumor growth under IFN-α treatment. Mechanistically, UBE2O ubiquitinates and degrades interferon-induced protein with tetratricopeptide repeats 3 (IFIT3), a key mediator of IFN-α signaling. High IFIT3 levels correlate with favorable IFN-α responses, underscoring UBE2O’s role in therapy resistance.

Huang et al. (2025) revealed that UBE2O modulates the DNA damage response (DDR) and radiation sensitivity in HCC [30]. UBE2O knockdown increases γ-H2AX foci (marking DNA damage), reduces Rad51 foci (impaired repair), and enhances radiosensitivity in vitro. In vivo, irradiated UBE2O-deficient tumors show attenuated growth, with reduced volume and weight. Mechanistically, ribosomal S6 kinase 2 (RSK2) phosphorylates UBE2O at Thr838, promoting its degradation and thereby enhancing radiosensitivity. This highlights the RSK2/UBE2O axis as a potential target for radiosensitization strategies.

#### 3.1.3. UBE2O Promotes Lung Cancer Progression and Radiation Resistance via Mxi1 Ubiquitination and Degradation

Lung cancer continues to be the leading cause of cancer-related deaths worldwide, with non-small cell lung cancer (NSCLC) accounting for approximately 85% of all cases [31,32]. Among NSCLC subtypes, lung adenocarcinoma is the most common, representing roughly 50% of cases [32]. Radiotherapy is a critical component of treatment across all stages of the disease and has been shown to significantly improve patient survival [33]. However, radioresistance remains a major challenge, driving local recurrence and distant metastasis. This underscores the urgent need to identify novel targets to enhance radiosensitivity.

Mxi1 downregulation is a hallmark of NSCLC [34]. Huang et al. elucidated this phenomenon by demonstrating UBE2O–Mxi1 interaction through co-immunoprecipitation (Co-IP) and glutathione S-transferase (GST) pull-down assays in vitro and in vivo [26]. Endogenous UBE2O bound Mxi1 in two lung cancer cell lines (H1299, A549). The silencing or knockout of UBE2O markedly increased Mxi1 protein levels, while UBE2O overexpression reduced them. Catalytically inactive UBE2O mutants (C1040S) and UBC domain deletion mutants (M2/M3) impaired Mxi1 ubiquitination. UBE2O knockout prolonged Mxi1 half-life, confirming UBE2O-mediated proteasomal degradation. Mass spectrometry identified lysine 46 (K46) of Mxi1 as the primary ubiquitination site targeted by UBE2O. Clinically, high UBE2O expression correlated with shorter overall survival, marking it as a poor prognostic indicator. Immunohistochemistry revealed an inverse correlation between UBE2O (high) and Mxi1 (low) expression in lung cancer tissues, consolidating their functional antagonism.

Elevated UBE2O expression accelerates lung cancer cell proliferation and tumor growth [26]. In vitro, UBE2O silencing suppressed cell proliferation and colony formation. In vivo, tumors with silenced UBE2O exhibited significantly slowed growth. Critically, UBE2O conferred radioresistance; UBE2O knockdown sensitized cells to radiation by impairing DNA damage repair, amplifying radiation-induced damage, and reducing clonogenic survival [26]. In xenograft models, combining UBE2O silencing with radiotherapy synergistically inhibited tumor growth compared to radiotherapy alone. Rescue experiments confirmed Mxi1 as the key downstream effector; Mxi1 knockdown in UBE2O-silenced cells restored proliferation, colony formation, and radioresistance.

#### 3.1.4. UBE2O Promotes Pancreatic Cancer Progression via BIN1 Degradation and c-Myc

Lin et al. demonstrated that UBE2O is highly expressed in pancreatic cancer tissues and serum exosomes, with elevated levels correlating with advanced pathological stage, lymph node metastasis, distant metastasis, and poor patient prognosis [35]. Functionally, UBE2O overexpression enhances pancreatic cancer cell proliferation, migration, and glycolytic activity in vitro, while UBE2O knockdown suppresses these phenotypes. In vivo, UBE2O overexpression accelerates tumor growth (increased volume/weight) in subcutaneous xenograft models, whereas UBE2O silencing inhibits tumor progression. Similarly, in lung metastasis models, UBE2O promotes metastatic dissemination, as evidenced by the increased presence of pulmonary nodules in overexpression groups and reduced metastasis upon knockdown.

The pro-tumorigenic effects of UBE2O are mediated through its interaction with BIN1 (Bcl-2-interacting protein 1), a tumor suppressor that antagonizes c-Myc transcriptional activity. In pancreatic cancer, BIN1 protein levels are markedly downregulated and inversely correlated with UBE2O expression. Lin et al. revealed that UBE2O binds to BIN1 via its BAR domain, functioning as an E2/E3 hybrid enzyme to mediate the K48-linked ubiquitination and proteasomal degradation of BIN1 [35]. This process is further amplified by circPDK1 (circular RNA-pyruvate dehydrogenase kinase 1), which acts as a scaffold to stabilize the UBE2O–BIN1 interaction, forming a ternary complex that accelerates BIN1 turnover.

The loss of BIN1 leads to the hyperactivation of c-MYC, a master regulator of proliferation and metastasis. UBE2O-driven BIN1 depletion relieves its suppression of c-MYC, thereby enhancing c-MYC transcriptional activity. This mechanism underpins UBE2O’s pro-tumorigenic effects, including metabolic reprogramming (e.g., elevated glycolysis) and metastatic progression. Consequently, the UBE2O–BIN1–c-Myc axis plays a pivotal role in tumor progression, highlighting UBE2O as a potential therapeutic target.

In conclusion, UBE2O drives pancreatic cancer progression by driving the ubiquitin-dependent degradation of BIN1 and thereby activating c-Myc-driven oncogenic pathways. Its overexpression corresponds to aggressive clinicopathological features and poor patient survival. UBE2O is thus a promising diagnostic, prognostic, and therapeutic target.

#### 3.1.5. UBE2O Promotes the Malignant Phenotypes of Thyroid Cancer by Modulating PLEKHG4 Stability

Yuan et al. investigated the regulatory role of UBE2O in thyroid cancer (TC) progression, and demonstrated that UBE2O modulates the stability of the Pleckstrin homology domain containing family G member 4 (PLEKHG4) to regulate the malignant behavior of thyroid cancer cells [36]. The detailed regulatory mechanisms are as follows.

Using bioinformatics analysis (via the IntAct and BioGRID platforms), the authors predicted an interaction between UBE2O and PLEKHG4, which was subsequently validated through co-immunoprecipitation (Co-IP) experiments. In HEK293T cells, Flag-tagged UBE2O was shown to interact with His-tagged PLEKHG4. This interaction was further confirmed in thyroid cancer cell lines CAL-62 and 8305C.

To determine whether UBE2O promotes the ubiquitination and degradation of PLEKHG4, the authors overexpressed or inhibited UBE2O in thyroid cancer cells and assessed PLEKHG4 protein levels. In CAL-62 cells overexpressing UBE2O, PLEKHG4 levels were significantly reduced, whereas in TPC-1 cells with UBE2O inhibition, PLEKHG4 levels were markedly increased. Treatment with the proteasome inhibitor MG132 revealed that the UBE2O-induced degradation of PLEKHG4 could be reversed, indicating that UBE2O mediates PLEKHG4 degradation via the proteasome pathway. Additionally, in HEK293T cells, UBE2O significantly enhanced the ubiquitination of PLEKHG4.

The authors further examined the effects of PLEKHG4 on cell proliferation, migration, invasion, and epithelial–mesenchymal transition (EMT) by overexpressing or knocking down PLEKHG4 in thyroid cancer cells. The overexpression of PLEKHG4 significantly promoted thyroid cancer cell proliferation, while its knockdown inhibited proliferation. Similarly, PLEKHG4 overexpression enhanced cell migration and invasion, whereas its knockdown suppressed these capabilities. The overexpression of PLEKHG4 also promoted the EMT process, as evidenced by the upregulation of mesenchymal markers (e.g., Vimentin and N-cadherin) and downregulation of epithelial markers (e.g., E-cadherin), while PLEKHG4 knockdown inhibited EMT.

The authors further explored the regulatory effects of PLEKHG4 on the activity of RhoGTPases, including Ras homolog family member A (RhoA), cell division cycle 42 (Cdc42), and Ras-related C3 botulinum toxin substrate 1 (Rac1). The overexpression of PLEKHG4 significantly increased the activity of RhoA, Cdc42, and Rac1, while its knockdown reduced their activity. Treatment with specific inhibitors of RhoA, Cdc42, and Rac1 reversed the enhanced proliferation, migration, and invasion induced by PLEKHG4 overexpression.

Integrating these findings, the authors proposed a regulatory mechanism involving the UBE2O/PLEKHG4/RhoGTPases axis in thyroid cancer progression. UBE2O ubiquitinates and degrades PLEKHG4, thereby regulating its protein stability. PLEKHG4, in turn, activates RhoGTPases (*RhoA*, *Cdc42*, and *Rac1*), which promote the proliferation, migration, invasion, and EMT process of thyroid cancer cells [36]. Collectively, UBE2O modulates PLEKHG4 stability, which subsequently influences RhoGTPase activity, thereby driving thyroid cancer progression [36].

#### 3.1.6. UBE2O Promotes Osteosarcoma Cell Proliferation and Tumor Growth via L3MBTL2 Protein Degradation

Osteosarcoma, a highly aggressive malignant bone tumor, is most commonly diagnosed in children and adolescents and remains the most prevalent primary malignant bone tumor in pediatric populations [37]. Despite over four decades of relatively stagnant treatment protocols—primarily surgical resection combined with neoadjuvant chemotherapy—the prognosis for osteosarcoma patients remains poor, with high rates of recurrence and metastasis [38,39]. These challenges underscore the critical need to elucidate the molecular mechanisms driving osteosarcoma progression and to identify novel therapeutic targets.

Zhong et al. demonstrated that UBE2O is markedly upregulated in osteosarcoma and correlates with adverse patient prognosis [39]. Employing in vitro and in vivo models, the authors established that UBE2O overexpression robustly enhances osteosarcoma cell proliferation and tumorigenesis, whereas UBE2O knockdown significantly attenuates these oncogenic processes.

Mechanistically, UBE2O mediates the multi-monoubiquitination of lethal 3 malignant brain tumor-like protein 2 (L3MBTL2), triggering its recognition and degradation via the proteasome [39]. By degrading L3MBTL2, UBE2O depletes this tumor suppressor protein, thereby compromising its tumor-suppressive function. L3MBTL2 undergoes phase separation to form nuclear condensates, which recruit members of the polycomb repressive complex 1.6 (PRC1.6) to repress the transcription of target genes, including interferon-induced protein with tetratricopeptide repeats 2 (IFIT2). The formation of these condensates is dependent on L3MBTL2’s polybasic region (PBR) and Pho-binding pocket. The UBE2O-mediated degradation of L3MBTL2 disrupts condensate formation, reducing PRC1.6 enrichment at the IFIT2 promoter and upregulating IFIT2 expression. IFIT2, a key mediator of the TNF/NF-κB signaling pathway, plays a critical role in osteosarcoma progression.

The degradation of L3MBTL2 leads to elevated IFIT2 expression, which is upregulated in osteosarcoma and correlates with tumor progression and poor prognosis. Increased IFIT2 levels activate the TNF/NF-κB pathway—a pivotal driver of osteosarcoma—resulting in enhanced cell proliferation, suppressed apoptosis, and heightened invasive and metastatic capacities [39]. Through these mechanisms, UBE2O eliminates the L3MBTL2-mediated suppression of osteosarcoma cell proliferation, thereby driving tumor growth.

In summary, UBE2O promotes osteosarcoma progression by degrading L3MBTL2, disrupting its nuclear condensates, derepressing IFIT2 transcription, and activating the TNF/NF-κB pathway. This study elucidates UBE2O’s oncogenic role in osteosarcoma, and identifies a potential therapeutic target for innovative treatment strategies.

#### 3.1.7. UBE2O Promotes Clear-Cell Renal Cell Carcinoma (ccRCC) Progression

Clinical studies demonstrate that elevated UBE2O expression in clear-cell renal cell carcinoma (ccRCC) is strongly associated with adverse clinical outcomes [40]. Mechanistically, UBE2O overexpression drives tumor progression and metastasis by stimulating cell proliferation, migration, and epithelial–mesenchymal transition (EMT), ultimately leading to reduced overall survival in patients [40]. Specifically, UBE2O accelerates ccRCC cell proliferation by promoting cell cycle progression, particularly the G1-to-S phase transition. Furthermore, UBE2O enhances the migratory and invasive properties of ccRCC cells through cytoskeletal reorganization and the modulation of adhesion molecules, including the downregulation of E-cadherin. Notably, UBE2O orchestrates EMT in ccRCC by ubiquitinating epithelial markers (e.g., E-cadherin) for proteasomal degradation while concurrently upregulating mesenchymal markers such as N-cadherin and Vimentin.

Lysine demethylase 1A (KDM1A), a histone demethylase that removes methyl groups from histones H3K4 and H3K9, is overexpressed in multiple cancers, including ccRCC, where it drives tumorigenesis. In ccRCC, KDM1A transcriptionally upregulates UBE2O expression. This regulatory relationship contributes to the poor prognosis associated with UBE2O overexpression in clinical cohorts.

#### 3.1.8. UBE2O Promotes Progression and EMT in Head and Neck Squamous Cell Carcinoma (HNSCC)

Head and neck squamous cell carcinoma (HNSCC), which encompasses malignancies of the oral cavity, pharynx, larynx, and salivary glands, ranks as the seventh most common cancer globally [41]. In 2018, approximately 890,000 new cases and 450,000 HNSCC-related deaths were reported worldwide [41]. Over the past few decades, its incidence has risen steadily, with projections suggesting an annual increase of ~30% by 2030 [42]. Regions such as South and Southeast Asia exhibit disproportionately high rates, largely attributed to betel quid chewing (often combined with tobacco) [43].

Chen et al. analyzed TCGA data to evaluate UBE2O expression in HNSCC and its clinical relevance [44]. Their findings demonstrate significantly elevated UBE2O mRNA levels in HNSCC tissues, with high UBE2O expression correlating with poorer survival outcomes. Functional studies have confirmed that UBE2O overexpression enhances HNSCC cell proliferation, migration, and invasion in vitro. Mechanistically, UBE2O induces EMT and potentiates TGFβ1-mediated EMT by modulating key markers (e.g., suppressing E-cadherin, upregulating N-cadherin/Vimentin) [44]. Consistent with this, xenograft models revealed that UBE2O knockout suppresses EMT, angiogenesis, and tumor growth in HNSCC.

In summary, UBE2O acts as an oncogenic factor in most solid tumors (Table 1).

### 3.2. The Function and Molecular Mechanism of UBE2O in Hematologic Malignancies

#### 3.2.1. UBE2O and Leukemia

The mixed-lineage leukemia (MLL) gene, located at chromosome 11q23, frequently undergoes chromosomal translocations that generate MLL fusion proteins (MLL chimeras). These fusion proteins are central to the pathogenesis of MLL-rearranged leukemias, including acute myeloid leukemia (AML) and acute lymphoblastic leukemia (ALL). These leukemia subtypes exhibit distinct clinical and biological features across pediatric and adult populations. Mechanistically, MLL fusion proteins drive leukemogenesis by constitutively activating genes involved in cell proliferation, differentiation, and survival. Notably, MLL fusion proteins exhibit high stability, enabling sustained oncogenic activity. In contrast, wild-type MLL protein, which is essential for normal hematopoiesis and transcriptional regulation, is typically downregulated in MLL-rearranged leukemias due to accelerated degradation.

UBE2O directly binds to an internal region of wild-type MLL (amino acids 1163–1482), which encompasses the breakpoint cluster region and the first PHD finger domain [45]. This interaction facilitates the UBE2O-mediated multi-monoubiquitination of wild-type MLL, marking it for proteasomal degradation and reducing its intracellular abundance [45]. Crucially, MLL fusion proteins lack this UBE2O-targeted domain due to truncation during chromosomal translocation, thereby evading degradation. Consequently, the persistence of stable MLL fusion proteins amplifies their oncogenic potential, while diminished wild-type MLL levels disrupt its tumor-suppressive roles in hematopoiesis and gene regulation [45]. This imbalance—enhanced stability of MLL fusion proteins coupled with the loss of wild-type MLL—establishes a dominant oncogenic state, driving leukemogenesis and disease progression.

The interleukin-1 (IL-1) signaling pathway, which is frequently hyperactivated in leukemia, potentiates UBE2O function via interleukin-1 receptor-associated kinase 4 (IRAK4). IRAK4 directly phosphorylates UBE2O, augmenting its capacity to ubiquitinate and degrade wild-type MLL. This phosphorylation event serves as a critical regulatory node, linking pro-inflammatory IL-1 signaling to MLL protein homeostasis.

In summary, UBE2O promotes leukemogenesis by selectively degrading wild-type MLL via multi-monoubiquitination, thereby tipping the balance toward oncogenic MLL fusion protein dominance. The IL-1/IRAK4 axis further amplifies this process through the post-translational activation of UBE2O. Targeting this axis may offer therapeutic opportunities in MLL-rearranged leukemia.

#### 3.2.2. UBE2O and Multiple Myeloma (MM)

In 2017, Xu et al. reported that UBE2O is highly expressed in normal bone marrow cells but is significantly downregulated in precursor lesions of multiple myeloma (MM), including monoclonal gammopathy of undetermined significance (MGUS) and smoldering multiple myeloma (SMM), as well as in MM cells [46]. This downregulation appears to be closely linked to MM initiation and progression, suggesting a tumor-suppressive role for UBE2O in MM [46]. The study further demonstrated that restoring UBE2O expression in MM xenograft tumors markedly delayed tumor growth and prolonged survival in tumor-bearing mice. These findings confirm that UBE2O functions as a tumor suppressor in MM [46].

The restoration of UBE2O expression in MM cells significantly reduced c-Maf protein levels and induced apoptosis [46]. Flow cytometry revealed that UBE2O increased apoptosis rates specifically in c-Maf-expressing MM cells, with no effect observed in c-Maf-deficient cells [46]. In a subcutaneous xenograft mouse model, UBE2O re-expression delayed MM tumor growth, correlating with c-Maf downregulation and the activation of apoptotic pathways [46].

Affinity purification coupled with tandem mass spectrometry (AP/MS) and co-immunoprecipitation confirmed a direct interaction between UBE2O and c-Maf, a master transcription factor in MM [46]. The overexpression of UBE2O in HEK293T cells enhanced c-Maf polyubiquitination and reduced its protein levels in a proteasome-dependent manner [46]. Critical lysine residues (K331 and K345) within c-Maf were identified as essential for UBE2O-mediated ubiquitination and degradation, as the mutation of these residues abolished these effects [46].

The c-Maf transcriptionally regulates downstream genes such as cyclin D2 (CCND2), integrin subunit beta 7 (ITGB7), and AMPK-related protein kinase 5 (ARK5), which drive MM cell proliferation, survival, and invasion [46]. By degrading c-Maf, UBE2O indirectly suppresses the expression of these oncogenic targets. For instance, the downregulation of CCND2 inhibits cell cycle progression, thereby curbing MM cell proliferation [46].

UBE2O specifically reduced c-Maf-dependent transcriptional activity. c-Maf activates the CCND2 promoter via its recognition element (MARE), but this activation was suppressed by UBE2O overexpression. Correspondingly, UBE2O overexpression led to CCND2 downregulation, a hallmark c-Maf target [46].

Mechanistically, UBE2O induced S or S/M phase arrest in c-Maf-expressing MM cells, but had no effect on c-Maf-deficient cells, aligning with c-Maf’s role in cell cycle regulation [46]. Similarly, UBE2O inhibited proliferation exclusively in c-Maf-expressing MM cells. In a nude mouse xenograft model, UBE2O re-expression significantly delayed MM tumor growth and extended survival, accompanied by elevated apoptosis in c-Maf-positive MM cells [46].

In summary, UBE2O suppresses MM progression by promoting the proteasomal degradation of c-Maf through site-specific polyubiquitination (K331/K345). This disrupts c-Maf-dependent oncogenic signaling (e.g., CCND2 activation), induces cell cycle arrest, and triggers apoptosis. These findings elucidate UBE2O’s tumor-suppressive role in MM and underscore its therapeutic potential.

In conclusion, UBE2O acts as an oncogenic factor in leukemia, but as a tumor suppressor in myeloma (Table 2). The dual role of UBE2O in cancer (oncogenic vs. tumor-suppressive) is likely closely related to tumor type-specific microenvironments, substrate selectivity, and differences in signaling pathways.

## 4. The Role of UBE2O in Alzheimer’s Disease (AD)

UBE2O, an E2–E3 hybrid enzyme, is involved in various physiological and pathological processes. In addition to its role in tumorigenesis and cancer progression, UBE2O also plays significant roles in several non-tumor diseases such as Alzheimer’s disease (AD) and metabolic diseases (Table 3).

AD is a common neurodegenerative disorder, the pathogenesis of which is closely related to the imbalance of protein homeostasis. The ubiquitin–proteasome system (UPS), which is crucial for maintaining protein homeostasis, declines in function with aging, and this decline is believed to be associated with the development of AD. UBE2O, as an E2–E3 hybrid enzyme, is an important component of the UPS. Cheng et al. systematically explored the function of UBE2O in Alzheimer’s disease (AD) and its molecular mechanisms through a series of experiments [47].

Using Western blot analysis, the authors detected the expression levels of UBE2O in various tissues (including brain, spinal cord, spleen, stomach, lung, intestine, liver, and kidney) of mice and found that UBE2O is highly expressed in the central nervous system (brain and spinal cord). The further detection of UBE2O expression in different brain regions (olfactory bulb, cortex, hippocampus, cerebellum, and striatum) revealed that its expression is highest in the cortex and hippocampus. By immunofluorescence co-staining, the authors found that UBE2O is highly expressed in neurons but not in glial cells. This finding was further confirmed by culturing primary neurons and glial cells.

The authors also detected the expression levels of UBE2O in the cortex and hippocampus of mice at different time points from postnatal day 7 (P7) to P240. The results show that UBE2O expression peaked at P17 in the cortex and P14 in the hippocampus, and then gradually decreased with age. In 6-month-old 5 × FAD transgenic mice (an AD model), UBE2O expression was significantly reduced in the cortex and hippocampus. Similarly, in SH-SY5Y cells overexpressing the Swedish mutant amyloid β-protein precursor (AβPP) (AβPPswe), UBE2O expression was significantly decreased (Table 3). Adding Aβ42 peptide to the culture medium of SH-SY5Y cells also led to a decrease in UBE2O expression. Neuronal death was significantly increased in cells overexpressing AβPPswe, while the overexpression of UBE2O significantly reduced neuronal death induced by AβPPswe.

In summary, these findings suggest that UBE2O may play an important role in the pathogenesis of AD (Table 3). Its reduction may promote neuronal death, while restoring UBE2O expression or function could potentially alleviate the pathological processes of AD (Table 3).

## 5. UBE2O and Metabolic Diseases

Obesity is closely associated with various metabolic diseases, such as type 2 diabetes, nonalcoholic fatty liver disease (NAFLD), and cardiovascular diseases. Insulin resistance, a core pathological factor in obesity and type 2 diabetes, is mainly manifested as the disruption of glucose and lipid metabolism in muscle, fat, and liver tissues. Although skeletal muscle plays a major role in insulin-stimulated glucose uptake, the mechanisms underlying insulin resistance in muscles are still unclear. AMPK is a key sensor of cellular energy and nutrient levels, and plays an important role in regulating metabolism and energy balance. UBE2O is preferentially expressed in metabolic tissues. Vila et al. aimed to explore the role of UBE2O in obesity and related metabolic disturbances [48].

The authors screened an siRNA library targeting 30 E2 ubiquitin-conjugating enzymes in human and mouse skeletal muscle myotubes, and found that UBE2O is a potent E2 enzyme that negatively regulates glucose transport. Using a high-fat diet (HFD)-induced obese mouse model, the authors detected the expression levels of UBE2O in skeletal muscle. The results show that UBE2O expression was significantly increased in the skeletal muscle of HFD-induced obese mice compared with normal diet-fed mice. The authors generated whole-body UBE2O knockout (*Ube2o^–/–^*) mice using CRISPR/Cas9 technology, and used wild-type (WT) mice as controls. The Ube2o^–/–^ and WT mice were fed an HFD or normal diet, and metabolic parameters such as body weight changes, food intake, fat mass, plasma lipid levels, liver weight, and hepatic fat content were monitored. The results show that *Ube2o^–/–^* mice gained less body weight, had reduced fat mass and improved plasma lipid levels, and were significantly protected against hepatic steatosis when fed an HFD. Indirect calorimetry was used to measure energy expenditure, respiratory exchange ratio (RER), and energy consumption in mice. The results indicate that *Ube2o^–/–^* mice had increased energy expenditure and a lower RER, indicating increased fat oxidation. Hyperinsulinemic–euglycemic clamp studies were conducted to assess insulin sensitivity. The results show that *Ube2o^–/–^* mice had significantly increased glucose infusion rates (GIR) and glucose disposal rates (GDR), indicating enhanced insulin sensitivity.

The authors generated skeletal muscle-specific UBE2O knockout (*Ube2oΔMus*) mice using the Cre-loxP system. The results show that Ube2oΔMus mice gained less body weight, had reduced fat mass, improved plasma lipid levels, and were significantly protected against hepatic steatosis when fed an HFD. Additionally, *Ube2oΔMus* mice had increased energy expenditure and enhanced insulin sensitivity. The authors also generated adipose tissue-specific (*Ube2oΔAdip*) and liver-specific (*Ube2oΔLiv*) UBE2O knockout mice. The results show that these mice did not exhibit significant differences in body weight gain, fat mass, plasma lipid levels, or liver fat content when fed an HFD, and their insulin sensitivity was not improved.

Co-immunoprecipitation experiments revealed a strong interaction between UBE2O and AMPKα2 in skeletal muscle. In vitro and in vivo ubiquitination assays showed that UBE2O, acting as an E2/E3 hybrid ubiquitin ligase, directly ubiquitinates AMPKα2 and promotes its proteasome-dependent degradation. In C2C12 myotubes, the overexpression or knockdown of UBE2O demonstrated that UBE2O inhibits insulin-stimulated glucose uptake and degrades AMPKα2 to suppress the phosphorylation of its substrates ACC and TBC1D1. In contrast, the knockdown of UBE2O increased glucose uptake, accompanied by elevated AMPKα2 expression and substrate phosphorylation.

The authors’ research indicates that UBE2O plays a significant role in obesity and metabolic syndrome. It targets AMPKα2 for ubiquitination and degradation in skeletal muscle, inhibiting the AMPK signaling pathway and thereby leading to insulin resistance and metabolic disorders (Table 3). The whole-body or skeletal muscle-specific knockout of UBE2O improves the metabolic status of obese mice, enhances insulin sensitivity, reduces fat accumulation, and protects against hepatic steatosis (Table 3). In contrast, the adipose tissue and liver-specific knockout of UBE2O has no significant impact on metabolic improvement (Table 3). Therefore, the UBE2O/AMPKα2 axis is an important regulator of metabolic homeostasis in skeletal muscle and a potential therapeutic target for diabetes and metabolic disorders (Table 3).

## 6. Strategies for Targeting UBE2O

Due to the role of UBE2O in various tumors, the development of UBE2O-specific inhibitors may become a new therapeutic strategy. The currently identified drugs targeting UBE2O mainly include arsenic trioxide and arborinine.

### 6.1. Arsenic Trioxide (ATO)

Arsenic trioxide (ATO) inhibits the ubiquitination function of UBE2O by forming covalent bonds with its cysteine residues, thereby blocking the binding of UBE2O to ubiquitin [49,50].

ATO has demonstrated significant inhibitory effects on various cancers through its ability to suppress the activity of UBE2O. In 2017, Vila et al. showed that ATO can inhibit UBE2O activity, restoring AMPKα2 expression and suppressing tumor growth and metabolic reprogramming in breast and prostate cancers [16]. ATO treatment reduced UBE2O-induced AMPKα2 ubiquitination, restoring AMPKα2 protein levels and inhibiting mTORC1 and HIF1α activity. In mouse models, ATO treatment effectively inhibited tumor growth, similar to UBE2O gene knockout.

In HCC, ATO treatment not only suppresses UBE2O protein levels, but also increases IFIT3 expression, thereby enhancing the efficacy of interferon-α [25]. In nude mouse models, the combination of ATO and interferon-α significantly reduced tumor volume, with higher ATO doses showing better effects.

ATO also exhibits inhibitory effects on osteosarcoma by cross-linking adjacent cysteines within the catalytic domain of UBE2O, thereby inhibiting its E3 ubiquitin ligase activity [39]. This treatment reduces the multi-monoubiquitination of L3MBTL2, stabilizing the protein and enhancing the formation of L3MBTL2 condensates. By stabilizing L3MBTL2, ATO suppresses IFIT2 gene expression, ultimately inhibiting osteosarcoma cell proliferation and tumor growth.

In lung cancer, ATO treatment significantly increases Mxi1 protein levels in a concentration- and time-dependent manner [26]. In nude mouse models, ATO (5 mg/kg) effectively inhibited tumor growth and enhanced the sensitivity of lung cancer cells to radiotherapy, as evidenced by increased DNA damage markers and reduced colony formation ability. When combined with radiotherapy, ATO further amplified the inhibitory effect on tumor growth, highlighting its potential as a therapeutic adjuvant.

### 6.2. Arborinine

Natural products provide a new perspective for cancer treatment due to their excellent compatibility and selectivity. Feng et al. revealed the role and molecular mechanisms of arborinine in clear-cell renal cell carcinoma (ccRCC). Arborinine is a selective and reversible inhibitor of KDM1A (LSD1) [40,51]. KDM1A is a histone demethylase that regulates gene transcription by removing methyl groups from histone H3K4 and H3K9. Molecular docking experiments showed that arborinine has a high binding prediction score with the KDM1A protein, indicating its ability to effectively bind and inhibit KDM1A activity. In ccRCC cell lines, treatment with arborinine significantly increased the levels of histone H3K4me1/2 and H3K9me1/2, further confirming the inhibitory effect of arborinine on KDM1A activity.

Through chromatin immunoprecipitation (ChIP) analysis and luciferase activity validation, UBE2O was identified as a transcriptional target downstream of KDM1A. In ccRCC cells, the expression of KDM1A is positively correlated with that of UBE2O, and the overexpression of UBE2O is associated with poor prognosis in ccRCC. Arborinine downregulates the expression of UBE2O by inhibiting the activity of KDM1A. Additionally, silencing KDM1A also significantly reduced the expression of UBE2O.

Arborinine significantly inhibits EMT in ccRCC cells, a key process by which tumor cells acquire invasiveness and metastatic potential. After treatment with arborinine, the expression of EMT-related markers (such as vimentin and N-cadherin) was significantly reduced, while the expression of E-cadherin increased. The study also measured the EMT index (EMTi) using a luciferase reporter gene assay and found that arborinine significantly reduced EMTi, an effect that was reversed by the overexpression of UBE2O. The study also indicated that arborinine exhibited significant inhibitory activity in drug-resistant ccRCC cell lines, suggesting its potential to overcome resistance to certain targeted therapies.

In conclusion, arborinine regulates the expression of UBE2O by inhibiting the activity of KDM1A, thereby inhibiting the proliferation, migration, and invasiveness of clear-cell renal cell carcinoma cells and reversing the EMT process. Additionally, arborinine may have potential in overcoming resistance to certain targeted therapies. These findings provide important theoretical support for the development of arborinine as a therapeutic agent for clear-cell renal cell carcinoma.

## 7. Conclusions and Future Prospects

### 7.1. Conclusions

**Figure 2:**
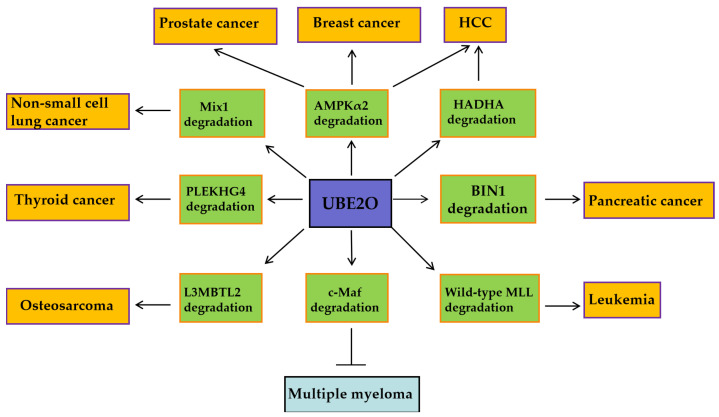
In cancer, UBE2O regulates primary substrates such as AMPKα2, MIXL1, HADHA, PLEKHG4, L3MBTL2, MLL, and c-Maf, among others. UBE2O is indicated by a purple box, while its downstream proteins are marked with green boxes. In tumors, UBE2O with pro-oncogenic functions is highlighted in yellow boxes, whereas UBE2O exhibiting tumor-suppressive roles is annotated with blue boxes.

**Figure 3:**
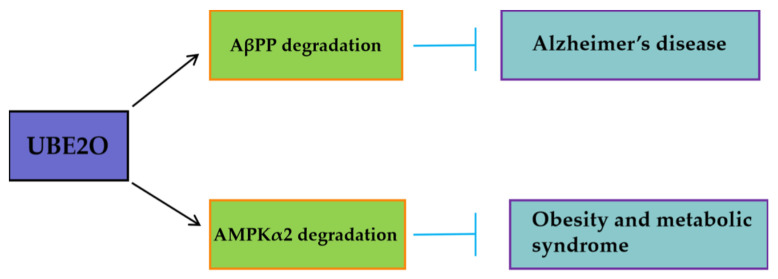
In non-neoplastic diseases, UBE2O targets key substrates such as AMPKα2 and AβPP. UBE2O is represented by a purple box, and its downstream proteins are denoted by green boxes. In non-neoplastic diseases, UBE2O with inhibitory functions is highlighted in blue boxes.

UBE2O exhibits pro-oncogenic activity in solid tumors and leukemia, while functioning as a tumor suppressor in multiple myeloma (MM). These context-dependent effects are mediated through its selective ubiquitination of distinct downstream substrates (Figure 2). In breast and prostate cancers, UBE2O promotes tumor cell proliferation and EMT by degrading AMPKα2 and activating the mTORC1 signaling pathway [16]. In lung cancer, UBE2O enhances tumor radioresistance by degrading Mxi1 and is associated with poor prognosis (Figure 2) [26]. Conversely, in multiple myeloma, UBE2O suppresses cell proliferation and survival by mediating the degradation of the transcription factor c-Maf, a key driver of the disease (Figure 2) [46]. Furthemore, beyond oncology, UBE2O may mitigate pathological processes in other diseases (Figure 3). By targeting AβPP (amyloid-β precursor protein) for degradation, UBE2O potentially reduces amyloid-β plaque formation, suggesting a protective role in Alzheimer’s disease (Figure 3) [47]. UBE2O could ameliorate obesity and metabolic syndrome by targeting AMPKα2 for degradation (Figure 3) [48].

Current UBE2O-targeting drugs include ATO and the natural alkaloid arborinine. ATO exerts anti-osteosarcoma effects by inhibiting UBE2O-mediated L3MBTL2 degradation, while arborinine blocks UBE2O’s degradation of substrates like AMPKα2 and demonstrates synergistic chemotherapeutic potential in breast and liver cancer models. These therapeutic strategies require further optimization and validation based on UBE2O’s tumor type-specific mechanisms.

### 7.2. Future and Prospects

As a key component of the UPS, UBE2O has demonstrated significant potential in recent research across fields such as cancer, AD, and metabolic disorders. Based on existing studies, its molecular mechanisms and disease associations have been gradually elucidated; however, numerous scientific questions remain to be explored. The following sections propose future research directions from three dimensions—mechanistic exploration, targeted intervention, and clinical translation.

In terms of mechanistic exploration, many questions remain to be addressed. First, the substrate specificity of UBE2O and its functional diversity in different cellular contexts need further investigation to clarify its precise role in various types of cancer. Second, the mechanisms by which UBE2O’s self-ubiquitination and dimerization regulate its enzymatic activity, as well as how these mechanisms influence its substrate recognition and ubiquitination processes, require deeper exploration. Additionally, the regulatory network of UBE2O and its interactions with other signaling pathways remain to be elucidated. For example, whether UBE2O synergizes with classical cancer pathways such as PI3K/AKT and Wnt/β-catenin warrants further investigation.

In terms of targeted intervention, ATO can enhance the tumor-suppressive function by inhibiting UBE2O activity, but its specificity and toxicity require optimization. Based on the crystal structure of UBE2O (e.g., the Pho-binding pocket), highly selective and specific inhibitors can be designed. In addition, CRISPR-Cas9 technology has been employed to screen UBE2O-associated pathway genes [1]. Future studies could combine gene editing with epigenetic drugs (e.g., histone modification inhibitors) to modulate UBE2O expression, thereby achieving precise regulation. Moreover, combination therapies that target UBE2O alongside other molecular targets may further enhance treatment efficacy and overcome drug resistance.

In terms of clinical translation, the following challenges must be addressed: the development of biomarkers, the elucidation of drug resistance mechanisms, and the integration of cross-disease studies. UBE2O exhibits a negative correlation with L3MBTL2 in osteosarcoma, and its high expression predicts poor prognosis [39]. Multicenter cohorts should be established to validate its universality as a diagnostic or prognostic biomarker and explore its application in liquid biopsies. Targeting UBE2O may trigger compensatory pathway activation (e.g., other E2 enzymes or ubiquitination bypass routes). Patient-derived organoid (PDO) models should be utilized to simulate resistance evolution and guide personalized therapeutic strategies. While UBE2O functions in tumors, AD, and metabolic abnormalities, its mode of action may vary across diseases. For instance, its potential role in antiviral immunity remains undefined. Interdisciplinary research is required to unravel the diversity of its pathophysiological functions.

As a key regulatory factor in the ubiquitination system, research on UBE2O is evolving from single-mechanism investigations to multidimensional network analyses. Future studies must integrate multi-omics, structural biology, and clinical data to uncover its systemic roles in diseases and facilitate the translation of targeted therapeutics from bench to bedside. Advancements in this field not only hold promise for novel therapies in oncology and immune disorders, but will also deepen the understanding of the complexity of the ubiquitination system, opening up novel avenues for precision medicine.

## Figures and Tables

**Table 1:** UBE2O and solid tumors.

Tumor Type	Substrates	Signal Pathway	Effects	References
Breast cancer	AMPKα2	AMPKα2/mTOR/HIF1α	Activation of mTOR pathway, increased cancer proliferation, promotion of EMT and metastasis, enhancement of CSC characteristics	[16,20]
Prostate cancer	AMPKα2	AMPKα2/mTOR/HIF1α	Activation of mTOR pathway, increased cancer proliferation, promotion of EMT and metastasis	[16]
Hepatocelluar carcimoma (HCC)	HADHA,AMPKα2	HADHA,AMPKα2/mTOR/HIF1α	HADHA ubiquitination and degradation, lipid metabolic reprogramming, liver cancer progression, UBE2O worsens the therapeutic effect of interferon-α	[22,23,24,25,30]
Non-small-cell lung cancer	Mxi1	Mxi1	Mxi1 ubiquitination and degradation, tumorigenesis and radiation resistance	[26]
Pancreatic cancer	BIN	BIN/c-Myc	BIN ubiquitination and degradation, the promotion of proliferation, migration, and glycolysis	[35]
Thyroid cancer	PLEKHG4	UBE2O/PLEKHG4/RhoGTPases	UBE2O promotes the proliferation, migration, invasion, and EMT process of thyroid cancer	[36]
Osteosarcoma	L3MBTL2	L3MBTL2/IFIT2,TNF/NF-κB pathway	UBE2O multi-monoubiquitinates and degrades L3MBTL2, UBE2O promotes tumor growth	[39]
Clear-Cell Renal Cell Carcinoma (ccRCC)	N/A	KDM1A/UBE2O	Increased cancer proliferation, migration and EMT	[40]
Head and neck squamous cell carcinoma (HNSCC)	N/A	E-cadherin, N-cadherin,Vimentin	Increased cancer proliferation, EMT and migration	[44]

**Table 2:** UBE2O and hematologic malignancies.

Tumor Type	Substrates	Signal Pathway	Effects	References
Leukemia	Wild-type MLL	IL-1/IRAK4/UBE2O/MLL	Wild-type MLL degradation, MLL fusion proteins stability, tumor progression	[45]
Multiple myeloma	c-Maf	c-Maf/CCND2, ITGB7, ARK5	The degradation of c-Maf, inhibited tumor growth	[46]

**Table 3:** UBE2O in several non-tumor diseases.

Tumor Type	Substrates	Signal Pathway	Effects	References
Alzheimer’s disease	AβPP	UBE2O/AβPP	The knockdown of UBE2O may promote neuronal death, while restoring UBE2O expression or function could potentially alleviate the pathological processes of AD.	[47]
Obesity and metabolic syndrome	AMPKα2	UBE2O/AMPKα2	UBE2O improves the metabolic status of obese mice, enhances insulin sensitivity, reduces fat accumulation, and protects against hepatic steatosis.	[48]

## Data Availability

Not applicable.

## Abbreviations

The following abbreviations are used in this manuscript:

ACC	Acetyl-CoA Carboxylase
AD	Alzheimer’s disease
ALL	Acute lymphoblastic leukemia
AML	Acute myeloid leukemia
AMPKα2	5′-AMP-activated protein kinase catalytic subunit alpha-2
AP/MS	Affinity purification coupled with tandem mass spectrometry
ATO	Arsenic trioxide
ATP	Adenosine triphosphate
AβPP	Amyloid β-protein precursor
BAP1	Breast cancer susceptibility gene 1(BRCA1)-associated protein 1
BIN1	Bcl-2 interacting protein 1
BMP7	Bone morphogenetic protein 7
BRCA1	Breast cancer susceptibility gene 1
CC	Coiled-coil
CCND2	Cyclin D2
ccRCC	Clear-cell renal cell carcinoma
Cdc42	Cell division cycle 42
CHIP	Chromatin immunoprecipitation
CHX	Cycloheximide
circPDK1	Circular RNA-pyruvate dehydrogenase kinase 1
c-Maf	v-maf musculoaponeurotic fibrosarcoma oncogene homolog
CSC	Cancer stem cell
CR	Conserved region
DDR	DNA damage response
DEN	Diethylnitrosamine
4E-BP1	Eukaryotic initiation factor 4E-binding protein 1
eIF4E	Eukaryotic initiation factor 4E
EMT	Epithelial–mesenchymal transition
ER	Estrogen receptor
F-6-P	Fructose-6-phosphate
G-6-P	Glucose-6-phosphate
GDR	Glucose disposal rates
GIR	Glucose infusion rates
GLUT1	Glucose transporter type 1
GST	Glutathione S-transferase
HADHA	Hydroxyacyl-CoA dehydrogenase
HCC	Hepatocellular carcinoma
HER2	Human epidermal growth factor receptor 2
HFD	High-fat diet
HIF1α	Hypoxia-inducible factor 1-alpha
HNSCC	Head and neck squamous cell carcinoma
γ-H2AX	Phospho-Histone H2A.X (Ser139)
IFIT3	Interferon induced protein with tetratricopeptide repeats 3
IFN	Interferon
IL-1	Interleukin-1
IRAK4	Interleukin-1 receptor-associated kinase 4
KDM1A	Lysine Demethylase 1A
L3MBTL2	Lethal 3 malignant brain tumor-like protein 2
MGUS	Monoclonal gammopathy of undetermined significance
MLL	Mixed-lineage leukemia
MM	Multiple myeloma
MMTV	The mouse mammary tumor virus
mTORC1	The mechanistic target of rapamycin complex 1
MTP	Mitochondrial trifunctional protein
Mxi1	MAX interactor 1
NACA	Nascent polypeptide-associated complex subunit alpha
NAFLD	Nonalcoholic fatty liver disease
NF-κB	Nuclear factor kappa-light-chain-enhancer of activated B cells
NLSs	Nuclear localization sequences
NSCLC	Non-small cell lung cancer
p97/VCP	Valosin-containing protein
PBR	polybasic region
PLEKHG4	Pleckstrin homology domain containing, family G member 4
PR	Progesterone receptor
PRC 1.6	Polycomb repressive complex 1.6
PyVT	Polyoma Virus middle T antigen
Rac1	Ras-related C3 botulinum toxin substrate 1
RAPA	Rapamycin
RER	Respiratory exchange ratio
RhoA	Ras homolog family member A
SLC25A3	Solute carrier family 25 member 3
SMM	Smoldering multiple myeloma
SREBP1	Sterol regulatory element binding protein 1
TBC1D1	TBC1 domain family member 1
TNBC	Triple-negative breast cancer
TRAF6	TNF receptor associated factor 6
TSC2	Tuberous sclerosis complex 2
UBC	Ubiquitin-conjugating
UBE2O	Ubiquitin-conjugating enzyme E2O
UPS	The ubiquitin–proteasome system
VEGFA	Vascular endothelial growth factor A
WT	Wild-type
